# Functions and mechanisms of lysine crotonylation

**DOI:** 10.1111/jcmm.14650

**Published:** 2019-09-01

**Authors:** Junhu Wan, Hongyang Liu, Jie Chu, Hongquan Zhang

**Affiliations:** ^1^ Department of Clinical Laboratory The First Affiliated Hospital of Zhengzhou University Zhengzhou China; ^2^ Department of Obstetrics and Gynecology The Third Affiliated Hospital of Zhengzhou University Zhengzhou China; ^3^ Department of Anatomy, Histology and Embryology Key Laboratory of Carcinogenesis and Translational Research Ministry of Education State Key Laboratory of Natural and Biomimetic Drugs Peking University Health Science Center Beijing China

**Keywords:** crotonylation, HCT, HDCR, PTM, reader

## Abstract

Lysine crotonylation is a newly discovered post‐translational modification, which is structurally and functionally different from the widely studied lysine acetylation. Recent advances in the identification and quantification of lysine crotonylation by mass spectrometry have revealed that non‐histone proteins are frequently crotonylated, implicating it in many biological processes through the regulation of chromatin remodelling, metabolism, cell cycle and cellular organization. In this review, we summarize the writers, erasers and readers of lysine crotonylation, and their physiological functions, including gene transcription, acute kidney injury, spermatogenesis, depression, telomere maintenance, HIV latency and cancer process. These findings not only point to the new functions for lysine crotonylation, but also highlight the mechanisms by which crotonylation regulates various cellular processes.

## 1 INTRODUCTION

Protein post‐translational modifications (PTMs), in which amino acid residues in a protein are covalently modified, have been increasingly recognized to play important roles in various biological pathways.^1^, ^2^ With the application of mass spectrometry‐based proteomics, novel histone PTMs have been documented, ranging from small chemical modifications (eg acetylation and phosphorylation) to the addition of complete proteins (eg ubiquitylation).^3^, ^4^ Post‐translational modification of proteins occurs in all living organisms. It governs many important cellular processes including the activation of enzymes, protein localization and protein degradation.^5^

Lysine is an amphipathic residue with a hydrophobic side chain. Acylation of lysine neutralizes the positive charge of the amino group and may change the conformation of proteins.^6^ According to the difference in hydrocarbon chain length, hydrophobicity and charge, the short‐chain lysine acylations include the well‐studied lysine acetylation and propionylation, butyrylation, 2‐hydroxyisobutyrylation, succinylation, malonylation, glutarylation, crotonylation and β‐hydroxybutyrylation.^7^, ^8^, ^9^, ^10^, ^11^, ^12^

Lysine crotonylation is a newly discovered histone PTM, which is specifically enriched at active gene promoters and potential enhancers in mammalian cell genomes.^11^ Crotonylation can be catalysed reversibly by protein crotonyltransferases and decrotonylases. The crotonylation of lysine was first identified on histones.^11^ Afterwards, more eukaryotic non‐histone proteins were identified as being crotonylated, and they were involved in cellular metabolism, cell cycle and cellular organization process.^13^, ^14^, ^15^, ^16^, ^17^, ^18^, ^19^, ^20^, ^21^, ^22^ In this review, we concentrate mainly on recent studies about lysine crotonylation and discuss its implications.

## 2 THE DISCOVERY OF LYSINE CROTONYLATION

**Figure 1:**
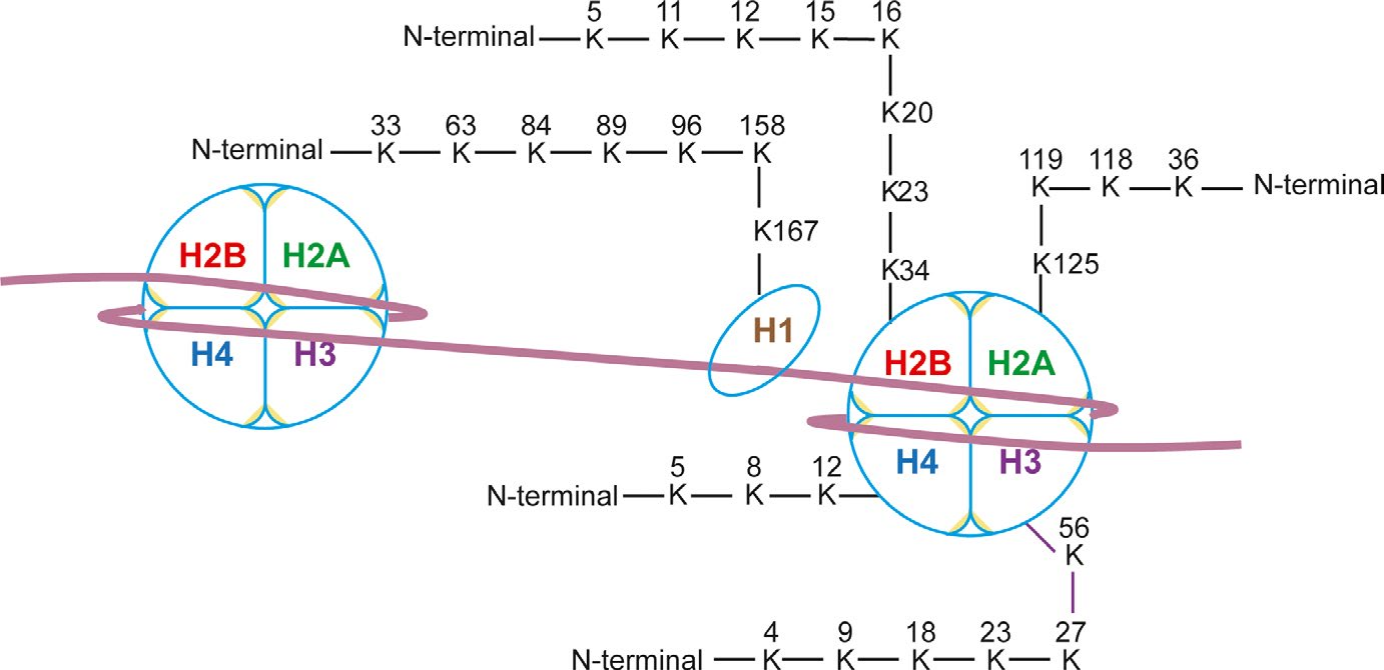
Illustrations of histone crotonylation sites in human. All reported lysine (K) crotonylation sites on histone H1, H2A, H2B, H3 and H4 are shown in different colours

Tan et al^11^ first reported protein lysine crotonylation in 2011. They used an integrated, mass spectrometry‐based proteomics approach, which takes advantage of in vitro propionylation, efficient peptide separation using isoelectric focusing (OFFGEL) and the high sensitivity of the LTQ Orbitrap Velos mass spectrometer to carry out a comprehensive analysis of histone PTMs. With this approach, they identified lysine crotonylation as a novel histone mark type. A total of 28 human histone peptides were found to have lysine crotonylation in this study (Figure 1). In addition, they also generated a specific anti‐crotonyl‐lysine antibody and applied it in corroborating Western blot and immunostaining experiments. Interestingly, they confirmed the existence of 19 crotonylation marks in HeLa cells using isotopic labelling with D4‐crotonate. The crotonyl group contains a unique C‐C π‐bond, which results in a rigid and planar configuration. Following the initial discovery of lysine crotonylation, the landscape of these modifications is rapidly expanding.

## 3 PROTEIN CROTONYLOME

Although initially identified on histones, lysine crotonylation has expanded to large number of non‐histone proteins. To characterize the global crotonylation proteome, the proteomic method based on sensitive immune‐affinity purification and high‐resolution liquid chromatography‐tandem (LC‐MS/MS) was applied to identify new crotonylated proteins and modification sites. The utilization of antibodies with high specificity to the crotonylated peptides involved in immunoprecipitation significantly improved the ability to enrich and identify crotonylated lysine residues. In recent years, several landmark studies have revealed dramatically improved number of crotonylated lysine residues and crotonylated proteins (Table 1), and these proteins play roles in nearly all cellular processes including chromatin remodelling, metabolism, cell cycle and cellular organization.^13^, ^14^, ^15^, ^16^, ^17^, ^18^, ^19^, ^20^, ^21^, ^22^ Crotonylome mapping provides an important resource for discovering novel properties and regulatory functions of crotonylation.

**Table 1:** Crotonylome studies discussed in this review

Species	Crotonylated peptides	Crotonylated proteins	Year	Refs
Human H1299 cells	2696	1024	2017	13
Human HeLa cells	558	453	2017	14
Human A549 cells	7765	2034	2017	15
Human HCT116 cells	816	392	2018	16
Human peripheral blood	1109	347	2018	17
Nicotiana tabacum	2044	637	2017	18
Zebrafish	557	218	2018	19
Rice	1265	690	2018	20
Rhodotorula mucilaginosa	1691	629	2018	21
Papaya fruit	5995	2120	2018	22

## 4 WRITERS OF LYSINE CROTONYLATION

**Figure 2:**
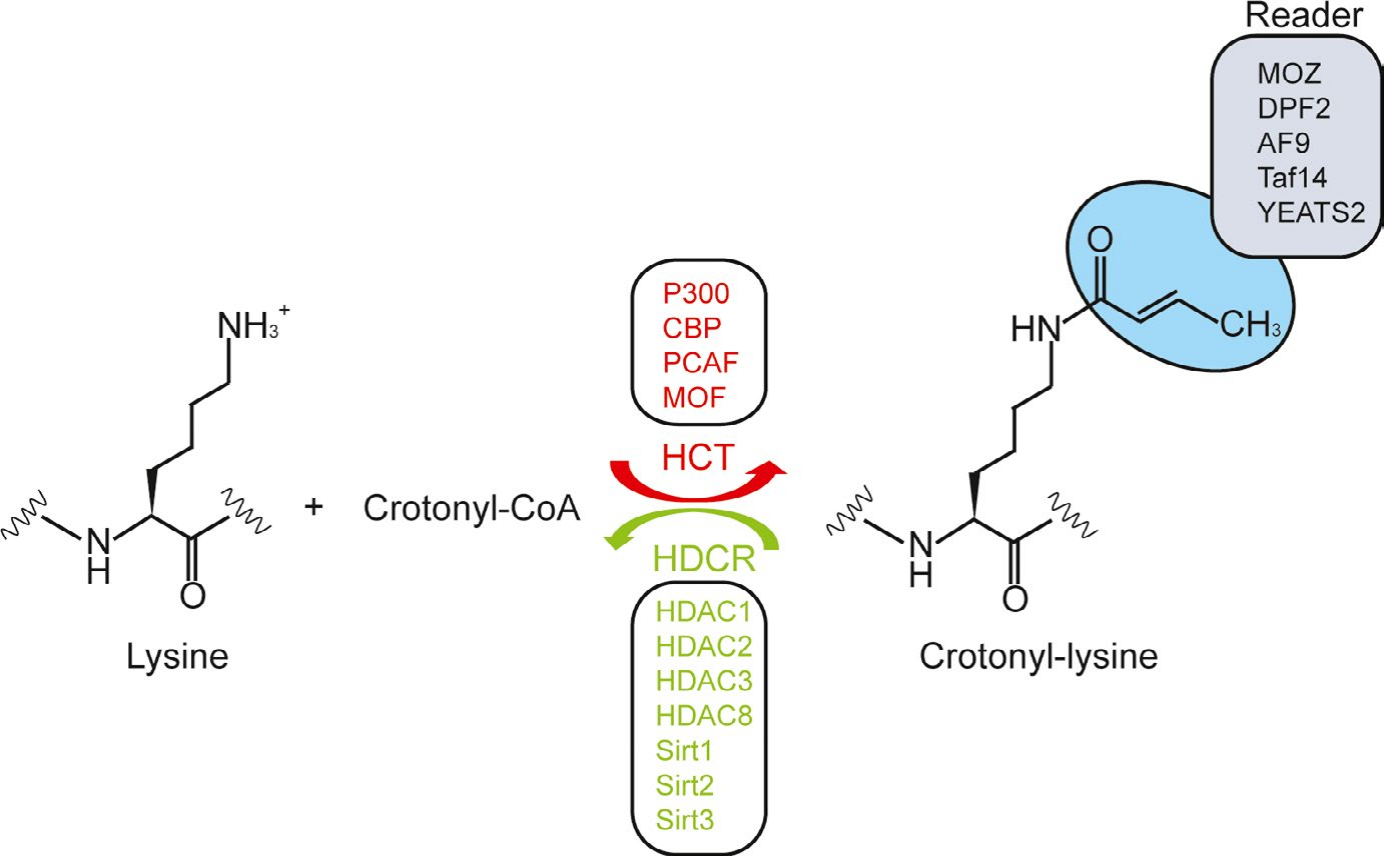
Protein crotonylation is balanced by HCT and HDCR, and recruits Readers. Protein crotonylation can be enzymatically catalysed by lysine crotonyltransferase (HCT) and removed by decrotonylase (HDCR). Crotonylations can also act as docking marks to recruit downstream readers

Lysine crotonylation is enzymatically regulated by the dynamic balance between crotonyltransferases and decrotonylases.^23^ The crotonyltransferases were colloquially termed writers, which catalyse the covalent modification of lysine crotonylation. The histone acetyltransferases (HATs) were also shown to have histone crotonyltransferase (HCT) activities (Figure 2; Table 2). There are three major HATs families, which can be categorized into three major families by their sequence and structural features: p300/CBP (CREB‐binding protein), GNAT (Gcn5‐related N‐acetyltransferases) and MYST (MOZ, Ybf2/Sas3, Sas2 and Tip60) families.^24^ The p300/CBP and MYST families are identified only in eukaryotic cells, while the GNAT family is present and conserved in all domains of life.

**Table 2:** Writers, erasers and readers discussed in this review

		Year	Refs
Writers	P300, CBP	2015	25
	CBP, MOF, PCAF	2017	13
	MOF	2017	26
Erasers	HDAC3	2012	28
	Sirt1, Sirt2	2013	29
	Sirt1, Sirt2, Sirt3	2014	30
	HDAC1, HDAC2, HDAC3, HDAC8	2017	31
	HDAC1 HDAC3	2017	13
	HDAC1 HDAC2	2018	32
Readers	MOZ, DPF2	2016	33
	AF9	2016	34
	Taf14	2016	35
	YEATS2	2016	36

Sabari et al^25^ first reported that p300 has both HAT and HCT activities. In the cell‐free assays, they demonstrated that p300‐catalysed histone crotonylation directly stimulated transcription to a greater degree than p300‐catalysed histone acetylation. Besides, the level of histone crotonylation was sensitive to changes in the cellular concentration of crotonyl‐CoA, due to p300's dual enzymatic activities. Afterwards, MOF was also identified to catalyse histone crotonylation, including histone H3 at lysine residues 4, 9, 18 and 23, and histone H4 at lysine 8 and 12.^26^ For the first time, Xu et al^13^ identified HAT can catalyse crotonylation on non‐histone proteins. In their study, they found non‐histone protein, NPM1, is strongly crotonylated by CBP and MOF, and moderately crotonylated by PCAF. However, the crotonylation of DEAD‐box RNA helicase, DDX5, can only be catalysed by CBP.

## 5 ERASERS OF LYSINE CROTONYLATION

Histone deacetylases (HDACs) were also reported to have histone decrotonylase (HDCR) activity (Figure 2; Table 2). The decrotonylases were colloquially termed erasers, which could remove the covalent modification of lysine crotonylation. Currently, two major families of KDACs have been divided into four classes: the NAD‐dependent sirtuin family (class III Sirt1‐7) and the zinc‐dependent Rpd3/Hda1 family (classes I [HDAC1, 2, 3 and 8], II [HDAC4, 5, 6, 7, 9 and 10] and IV [HDAC11]).^27^

HDAC3 was firstly reported to exhibit HDCR activity in vitro. By utilizing a collection of fluorogenic substrates, HDAC3‐NCoR1 was exhibited decrotonylase activity with a catalytic efficiency that is comparable to the deacetylase activity of other KDAC isoforms.^28^ After analysing the deacylase activity of Sirt1 through SIRT7 using catalytic amounts of enzyme and different expansive panel of acylated H3K9 peptides, Sirt1 and Sirt2 were also indicated act as efficient decrotonylases.^29^ With the application of an optimized CLASPI approach to comprehensively profile ‘eraser’ enzymes, Bao et al^30^ identified human Sirt1, Sirt2 and Sirt3 as decrotonylases in vitro, and using X‐ray crystallography, they examined the molecular basis of how the enzymes recognize crotonylated group. Afterwards, class I HDACs and Sirt1 were proved as active HDCR enzymes,^31^ by screening ectopically expressed HDAC and Sirt family proteins in HeLa cells, with immunofluorescence staining using an anti‐crotonyl‐lysine specific antibody. Kelly et al^32^ found that knock‐down HDAC1/2 in embryonic stem (ES) cells increased global histone crotonylation levels and caused a great reduction in total decrotonylase activity. Xu et al^13^ found that HDAC1 and HDAC3 decrotonylate NPM1, which can be reversed upon TSA treatment.

## 6 READERS OF LYSINE CROTONYLATION

Double PHD finger (DPF), bromodomain and YEATS are three major classes of acetylation and non‐acetyl acylation readers.^6^ Histone crotonylations can act as docking marks to recruit downstream readers (Figure 2; Table 2). Using ChIP‐qPCR and immunofluorescence assays, Xiong et al^33^ suggested the colocalization of MOZ and H3K14 crotonylation, and they also indicated that DPF domains of MOZ and DPF2 maintained high affinity for crotonylation at H3K14. A recent study reported that the binding affinity between AF9 YEATS domain and crotonyl‐lysine was higher than that to acetyl‐lysine.^34^ Furthermore, using heteronuclear NMR spectroscopy, they determined a 3D solution structure of the AF9 YEATS domain bound to an H3 crotonylated at Lys 18 (H3K18cr) peptide. Afterwards, Taf14 YEATS domain was identified as a reader of crotonyl‐lysine binding to histone H3 crotonylated at Lys 9 (H3K9cr) via a unique π‐π‐π stacking mechanism.^35^ Using a modified histone peptide array, Zhao et al^36^ found that YEATS2 bound to acylated histone peptides with the best preference for histone H3 crotonylated at Lys 27 (H3K27cr) peptide.

## 7 FUNCTIONAL ROLES OF LYSINE CROTONYLATION

### 7.1 Regulation of gene transcription

Tan et al^11^ firstly revealed the function of histone crotonylation in regulating gene transcription. They found histone crotonylation specifically labelled the enhancers and transcription starting site of active genes in human somatic and mouse male germ cell genomes. In addition, histone crotonylation was enriched on sex chromosomes and specifically marked X‐linked genes that escaped sex chromosome inactivation in male germinal cells immediately following meiosis. A recent study reported p300‐mediated histone crotonylation stimulates gene transcription to a greater degree than histone acetylation.^37^ With a cell‐based model of transcriptional activation, they also suggested different level of cellular crotonyl‐CoA concentration led the changes in histone crotonylation levels flanking the regulatory elements of activated genes and was correlated with gene expression. By using the novel CBP/p300 mutants (p300 I1395G and CBP I1432G) with deficient histone acetyltransferase but competent HCT activity, Liu et al^26^ demonstrated CBP/p300 can promote transcriptional activation in the absence of HAT and presence of HCT activity. Afterwards, their team colleagues found selective HDCR in mammalian cells correlates with a broad transcriptional repression, by using novel HDAC1 and HDAC3 mutants (HDAC1/3‐VRPP) with impaired HDAC but intact HDCR activity.^31^

### 7.2 Regulation of acute kidney injury

Acute kidney injury (AKI) is a potentially lethal condition with no available therapy beyond replacement of renal function.^38^ Ruiz‐Andres et al^39^ studied histone crotonylation level of acute kidney injury induced by folic acid or cisplatin, in murine proximal tubular cells and kidneys from mice. Furthermore, they indicated the histone crotonylation level of kidney tissue was increased during AKI. Beside, ChIP‐seq assays revealed histone crotonylation was enriched at the genes encoding the mitochondrial biogenesis regulator PGC‐1α and the sirtuin‐3 decrotonylase. When the crotonylation level was increased by adding crotonate in cultured tubular cells or in the kidneys in vivo, the expression of PGC‐1α and sirtuin‐3 was increased. In addition, crotonate administration protected from experimental AKI and prevented the decrease in renal function. For the first time, they suggested increased histone crotonylation level might have a beneficial effect on AKI.

### 7.3 Regulation of spermatogenesis

Genome‐wide removal of histones from chromatin and their replacement is a unique epigenetic event during spermatogenesis.^40^ Liu et al^41^ showed that chromodomain Y‐like transcription corepressor CDYL acted as a crotonyl‐CoA hydratase to convert crotonyl‐CoA to β‐hydroxybutyryl‐CoA, as a result destroying crotonyl‐CoA for histone crotonylation reaction. They also indicated histone crotonylation was associated with the reactivation of the sex chromosome‐linked genes of round spermatids. Using ChIP‐quantitative PCR, they suggested histone crotonylation levels in round spermatids cells were significantly higher than those in spermatocytes cells, indicating histone crotonylation levels during spermatogenesis are regulated by various complex mechanisms including both enzymatic and nonenzymatic reactions.

### 7.4 Regulation of depression

Histone crotonylation was found decreased in the medial prefrontal cortex of susceptible rodents exposed to chronic social defeat stress.^42^ In addition, when knocking down CDYL in prelimbic cortex, the histone crotonylation was increased. Using ChIP‐quantitative PCR, this finding also demonstrated that CDYL inhibits VGF transcription by its dual influence on histone crotonylation and H3K27me3 of VGF promoter. For the first time, the relationship between crotonylation and depression was identified, and CDYL‐mediated histone crotonylation may play an important role in regulating stress‐induced depressive behaviours.

### 7.5 Regulation of telomere maintenance

Telomere elongation with increasing passage depends on the mechanisms of both telomerase and recombination‐based alternative lengthening of telomeres.^43^ When crotonylation induced by crotonic acid, Zscan4 was found activated, and T‐SCE level increased, which maintained telomeres and reduced telomere damage during chemical induction.^44^ In addition, chemically induced pluripotent stem cells' (CiPSC) clone formation at stage II during middle induction was improved by crotonylation, indicating that extraembryonic endoderm state is primed for action by crotonic acid.

### 7.6 Regulation of HIV latency

Latent HIV reservoirs in the host are established early before viral infection.^45^, ^46^ ACSS2, the crotonyl‐CoA‐producing enzyme acyl‐CoA synthetase short‐chain family member 2, was identified to influence HIV replication and viral latency by regulating histone crotonylation at HIV long‐terminal repeat.^47^ After histone crotonylation was induced by ACSS2 in vitro and ex vivo, HIV latency was disrupted. Furthermore, when inhibiting histone crotonylation with suppressing ACSS2, latent HIV reactivation was dampened, suggesting a potential role of histone decrotonylation in HIV latency establishment. For the first time, this finding linked maintenance of viral latency to histone crotonylation by ACSS2 at the HIV long‐terminal repeat.

### 7.7 Regulation of cancer

Cancer is a life‐threatening malignancy that has become a global healthcare problem.^48^, ^49^, ^50^ Using immunohistochemical methods, we recently reported that levels of crotonylation in eight types of cancer.^51^ We found the expression level of crotonylation was down‐regulated in liver, stomach and kidney carcinomas, and up‐regulated in thyroid, oesophagus, colon, pancreas and lung carcinomas, suggesting that crotonylation may modulate different cancer progression. Besides, lysine crotonylation was identified involving in hepatoma cell motility and proliferation. For the first time, we indicated that status of lysine crotonylation may represent an important type of post‐translational modifications accounting for cancer progression.

## 8 CONCLUSIONS

Lysine crotonylation is recently identified as a novel evolutionarily conserved histone PTM.^11^ The recent discovery of a large panel of new histone PTMs, including lysine crotonylation, may change our vision of epigenome.^6^, ^52^ Lysine crotonylation is involved in many pathways that regulate diverse cellular functions ranging from gene expression to telomere maintenance (Table 3). It would be interesting in future to determine how their functions are mechanistically regulated by crotonylation.

**Table 3:** Functional roles of lysine crotonylation discussed in this review

Functional roles of lysine crotonylation	In vivo, ex vivo or cell culture studies	Descriptive or interventional studies	The model studied	Year	Refs
Regulation of gene transcription	In vivo	Descriptive study	Meiotic and postmeiotic male germ cells and tissues	2011	11
	Cell culture	Interventional study	293T cells	2017	26
	Cell culture	Interventional study	HeLa cells	2017	31
	Ex vivo and cell culture	Interventional study	Cell‐free system and LPS induced inflammatory response in the macrophage cell line RAW 264.7	2018	37
Regulation of acute kidney injury	In vivo and cell culture	Interventional study	Cultured murine proximal tubular cells and kidneys from mice with AKI	2016	39
Regulation of spermatogenesis	In vivo	Descriptive study	Mouse testes	2017	41
Regulation of depression	In vivo	Interventional study	Mice model	2018	42
Regulation of telomere maintenance	Cell culture	Interventional study	MEF cells	2018	44
Regulation of HIV latency	Cell culture	Interventional study	J‐Lat A1 and CD4^+^ T cells	2018	47
Regulation of cancer	In vivo and cell culture	Interventional study	HCC tissues and human hepatoma‐derived cell	2019	51

The developments in high‐resolution LC‐MS/MS approaches have enabled the crotonylome‐wide mapping of lysine crotonylation.^4^ Thus, future crotonylome studies should be well designed. Identification of crotonylation on non‐histone proteins could expand our understanding of how non‐histone proteins are involved in diverse cellular functions and signalling pathways, and may facilitate the clarification of the precise modulation on protein functions.

In general, one protein may occur simultaneously multiple PTMs.^53^ Indeed, recent years gradually intensifying research on crotonylation and many publications were dedicated to only one of the many possible lysine crotonylations. Even for crotonylation, most crotonylated proteins have multiple crotonylated lysine residues. Therefore, the crosstalk between different PTMs and their contributions in regulating protein function should be investigated. Human disease in which multiple factors may be involved, is a complex phenotype and cannot be easily interpreted by analysing a single type PTM.^54^ Whether different PTMs could be functionally grouped or specific signalling to chromatin depends on each PTMs. The hypothesis would imply that diverse PTMs may cooperate with each other to provide a wealth of regulatory potential.

Over the past years, we have witnessed the tremendous advances in understanding of the mechanisms and cellular functions on protein crotonylation. However, it still exists many limitations in the published studies. Despite a growing understanding of crotonylation function in regulating diverse physiological function, it remains unclear how such biochemical changes occur and whether they play crucial roles in more disease progression. Besides, current crotonylation studies have mainly focused on a small number of histones, limiting the ability to clarify the relationship between crotonylation and human physiological processes. Moreover, the number of writers, erasers and readers of lysine crotonylation found so far is still very limited, more studies on the discovery and identification of writers, erasers and readers will be helpful for understanding the function of crotonylation.

In the further research, we should focus on how human physiological processes and related diseases mechanistically regulated by crotonylation. Beside, more studies should search for understanding the role of crotonylation in non‐histones. We can also discovery more sites about lysine crotonylation, including the mechanisms by which they occur. Therefore, future studies are needed to uncover the effects of crotonylation on regulating protein functions and to interpret the underlying mechanisms behind protein crotonylation's ability to modulate diverse physiological and pathological processes.

## CONFLICT OF INTEREST

The authors declared no conflict of interest.

## AUTHOR CONTRIBUTIONS

JHW and HQZ provided direction and guidance throughout the preparation of this manuscript. JHW draw the graph. HYL and JC collected and prepared the related literature. All authors have read and approved the final manuscript.

## Data Availability

Data will be available upon reasonable request.

## References

[1] Verdin E , Ott M . 50 years of protein acetylation: from gene regulation to epigenetics, metabolism and beyond. Nat Rev Mol Cell Biol. 2015;16:258‐264. 25549891 10.1038/nrm3931

[2] Kouzarides T . Chromatin modifications and their function. Cell. 2007;128:693‐705. 17320507 10.1016/j.cell.2007.02.005

[3] Huang H , Sabari BR , Garcia BA , Allis CD , Zhao Y . SnapShot: histone modifications. Cell. 2014;159:458‐458.e1. 25303536 10.1016/j.cell.2014.09.037 PMC4324475

[4] Fu J , Wu M , Liu X . Proteomic approaches beyond expression profiling and PTM analysis. Anal Bioanal Chem. 2018;410:4051‐4060. 29637251 10.1007/s00216-018-1021-y

[5] Dutta A , Abmayr SM , Workman JL . Diverse activities of histone acylations connect metabolism to chromatin function. Mol Cell. 2016;63:547‐552. 27540855 10.1016/j.molcel.2016.06.038 PMC5298895

[6] Sabari BR , Zhang D , Allis CD , Zhao Y . Metabolic regulation of gene expression through histone acylations. Nat Rev Mol Cell Biol. 2017;18:90‐101. 27924077 10.1038/nrm.2016.140 PMC5320945

[7] Dai L , Peng C , Montellier E , et al. Lysine 2‐hydroxyisobutyrylation is a widely distributed active histone mark. Nat Chem Biol. 2014;10:365‐370. 24681537 10.1038/nchembio.1497

[8] Chen Y , Sprung R , Tang YI , et al. Lysine propionylation and butyrylation are novel post‐translational modifications in histones. Mol Cell Proteomics. 2007;6:812‐819. 17267393 10.1074/mcp.M700021-MCP200 PMC2911958

[9] Xie Z , Dai J , Dai L , et al. Lysine succinylation and lysine malonylation in histones. Mol Cell Proteomics. 2012;11:100‐107. 22389435 10.1074/mcp.M111.015875 PMC3418837

[10] Tan M , Peng C , Anderson K , et al. Lysine glutarylation is a protein posttranslational modification regulated by SIRT5. Cell Metab. 2014;19:605‐617. 24703693 10.1016/j.cmet.2014.03.014 PMC4108075

[11] Tan M , Luo H , Lee S , et al. Identification of 67 histone marks and histone lysine crotonylation as a new type of histone modification. Cell. 2011;146:1016‐1028. 21925322 10.1016/j.cell.2011.08.008 PMC3176443

[12] Xie Z , Zhang D , Chung D , et al. Metabolic regulation of gene expression by histone lysine beta‐hydroxybutyrylation. Mol Cell. 2016;62:194‐206. 27105115 10.1016/j.molcel.2016.03.036 PMC5540445

[13] Xu W , Wan J , Zhan J , et al. Global profiling of crotonylation on non‐histone proteins. Cell Res. 2017;27:946‐949. 28429772 10.1038/cr.2017.60 PMC5518986

[14] Wei W , Mao A , Tang B , et al. Large‐scale identification of protein crotonylation reveals its role in multiple cellular functions. J Proteome Res. 2017;16:1743‐1752. 28234478 10.1021/acs.jproteome.7b00012

[15] Wu Q , Li W , Wang C , et al. Ultradeep lysine crotonylome reveals the crotonylation enhancement on both histones and nonhistone proteins by SAHA treatment. J Proteome Res. 2017;16:3664‐3671. 28882038 10.1021/acs.jproteome.7b00380

[16] Huang H , Wang DL , Zhao Y . Quantitative crotonylome analysis expands the roles of p300 in the regulation of lysine crotonylation pathway. Proteomics. 2018;18:e1700230. 29932303 10.1002/pmic.201700230 PMC6420807

[17] Chen W , Tang D , Xu Y , et al. Comprehensive analysis of lysine crotonylation in proteome of maintenance hemodialysis patients. Medicine. 2018;97:e12035. 30212933 10.1097/MD.0000000000012035 PMC6156053

[18] Sun H , Liu X , Li F , et al. First comprehensive proteome analysis of lysine crotonylation in seedling leaves of Nicotiana tabacum. Sci Rep. 2017;7:3013. 28592803 10.1038/s41598-017-03369-6 PMC5462846

[19] Kwon OK , Kim SJ , Lee S . First profiling of lysine crotonylation of myofilament proteins and ribosomal proteins in zebrafish embryos. Sci Rep. 2018;8:3652. 29483630 10.1038/s41598-018-22069-3 PMC5827021

[20] Liu S , Xue C , Fang Y , et al. Global involvement of lysine crotonylation in protein modification and transcription regulation in rice. Mol Cell Proteomics. 2018;17:1922‐1936. 30021883 10.1074/mcp.RA118.000640 PMC6166680

[21] Yang Q , Li Y , Apaliya MT , et al. The response of *Rhodotorula mucilaginosa* to patulin based on lysine crotonylation. Front Microbiol. 2018;9:2025. 30233516 10.3389/fmicb.2018.02025 PMC6129574

[22] Liu K , Yuan C , Li H , et al. A qualitative proteome‐wide lysine crotonylation profiling of papaya (*Carica papaya* L.). Sci Rep. 2018;8:8230. 29844531 10.1038/s41598-018-26676-y PMC5974297

[23] Zhao S , Zhang X , Li H . Beyond histone acetylation‐writing and erasing histone acylations. Curr Opin Struct Biol. 2018;53:169‐177. 30391813 10.1016/j.sbi.2018.10.001

[24] Narita T , Weinert BT , Choudhary C . Functions and mechanisms of non‐histone protein acetylation. Nat Rev Mol Cell Biol. 2019;20:156‐174. 30467427 10.1038/s41580-018-0081-3

[25] Sabari B , Tang Z , Huang HE , et al. Intracellular crotonyl‐CoA stimulates transcription through p300‐catalyzed histone crotonylation. Mol Cell. 2015;58:203‐215. 25818647 10.1016/j.molcel.2015.02.029 PMC4501262

[26] Liu X , Wei W , Liu Y , et al. MOF as an evolutionarily conserved histone crotonyltransferase and transcriptional activation by histone acetyltransferase‐deficient and crotonyltransferase‐competent CBP/p300. Cell Discov. 2017;3:17016. 28580166 10.1038/celldisc.2017.16 PMC5441097

[27] Ren J , Sang Y , Lu J , Yao YF . Protein acetylation and its role in bacterial virulence. Trends Microbiol. 2017;25:768‐779. 28462789 10.1016/j.tim.2017.04.001

[28] Madsen AS , Olsen CA . Profiling of substrates for zinc‐dependent lysine deacylase enzymes: HDAC3 exhibits decrotonylase activity in vitro. Angew Chem Int Ed Engl. 2012;51:9083‐9087. 22890609 10.1002/anie.201203754

[29] Feldman JL , Baeza J , Denu JM . Activation of the protein deacetylase SIRT6 by long‐chain fatty acids and widespread deacylation by mammalian sirtuins. J Biol Chem. 2013;288:31350‐31356. 24052263 10.1074/jbc.C113.511261 PMC3829447

[30] Bao X , Wang YI , Li X , et al. Identification of 'erasers' for lysine crotonylated histone marks using a chemical proteomics approach. Elife. 2014;3:e02999. 10.7554/eLife.02999 PMC4358366 25369635

[31] Wei W , Liu X , Chen J , et al. Class I histone deacetylases are major histone decrotonylases: evidence for critical and broad function of histone crotonylation in transcription. Cell Res. 2017;27:898‐915. 28497810 10.1038/cr.2017.68 PMC5518989

[32] Kelly R , Chandru A , Watson PJ , et al. Histone deacetylase (HDAC) 1 and 2 complexes regulate both histone acetylation and crotonylation in vivo. Scientific Reports. 2018;8:14690. 30279482 10.1038/s41598-018-32927-9 PMC6168483

[33] Xiong X , Panchenko T , Yang S , et al. Selective recognition of histone crotonylation by double PHD fingers of MOZ and DPF2. Nat Chem Biol. 2016;12:1111‐1118. 27775714 10.1038/nchembio.2218 PMC5253430

[34] Zhang Q , Zeng L , Zhao C , Ju Y , Konuma T , Zhou MM . Structural Insights into histone crotonyl‐lysine recognition by the AF9 YEATS domain. Structure. 2016;24:1606‐1612. 27545619 10.1016/j.str.2016.05.023 PMC5014688

[35] Andrews FH , Shinsky SA , Shanle EK , et al. The Taf14 YEATS domain is a reader of histone crotonylation. Nat Chem Biol. 2016;12:396‐398. 27089029 10.1038/nchembio.2065 PMC4871749

[36] Zhao D , Guan H , Zhao S , et al. YEATS2 is a selective histone crotonylation reader. Cell Res. 2016;26:629‐632. 27103431 10.1038/cr.2016.49 PMC4856769

[37] Sabari BR , Tang Z , Huang HE , et al. Intracellular Crotonyl‐CoA stimulates transcription through p300‐catalyzed histone crotonylation. Mol Cell. 2018;69:533. 10.1016/j.molcel.2018.01.013 PMC5808432 29395068

[38] Berger K , Moeller MJ . Mechanisms of epithelial repair and regeneration after acute kidney injury. Semin Nephrol. 2014;34:394‐403. 25217268 10.1016/j.semnephrol.2014.06.006

[39] Ruiz‐Andres O , Sanchez‐Niño MD , Cannata‐Ortiz P , et al. Histone lysine crotonylation during acute kidney injury in mice. Dis Model Mech. 2016;9:633‐645. 27125278 10.1242/dmm.024455 PMC4920150

[40] Meistrich ML , Mohapatra B , Shirley CR , Zhao M . Roles of transition nuclear proteins in spermiogenesis. Chromosoma. 2003;111:483‐488. 12743712 10.1007/s00412-002-0227-z

[41] Liu S , Yu H , Liu Y , et al. Chromodomain protein CDYL acts as a crotonyl‐CoA hydratase to regulate histone crotonylation and spermatogenesis. Mol Cell. 2017;67(853–66):e5. 10.1016/j.molcel.2017.07.011 28803779

[42] Liu Y , Li M , Fan M , et al. Chromodomain Y‐like protein‐mediated histone crotonylation regulates stress‐induced depressive behaviors. Biol Psychiat. 2019;85:635‐649. 30665597 10.1016/j.biopsych.2018.11.025

[43] Liu L . Linking telomere regulation to stem cell pluripotency. Trends Genet. 2017;33:16‐33. 27889084 10.1016/j.tig.2016.10.007

[44] Fu H , Tian C‐L , Ye X , et al. Dynamics of telomere rejuvenation during chemical induction to pluripotent stem cells. Stem Cell Reports. 2018;11:70‐87. 29861168 10.1016/j.stemcr.2018.05.003 PMC6066961

[45] Dandekar S . Pathogenesis of HIV in the gastrointestinal tract. Current HIV/AIDS Reports. 2007;4:10‐15. 17338855 10.1007/s11904-007-0002-0

[46] Somsouk MA , Estes JD , Deleage C , et al. Gut epithelial barrier and systemic inflammation during chronic HIV infection. AIDS. 2015;29:43‐51. 25387317 10.1097/QAD.0000000000000511 PMC4444362

[47] Jiang G , Nguyen D , Archin NM , et al. HIV latency is reversed by ACSS2‐driven histone crotonylation. J Clin Invest. 2018;128:1190‐1198. 29457784 10.1172/JCI98071 PMC5824862

[48] Wan J , Liu H , Yang L , Ma L , Liu J , Ming L . JMJD6 promotes hepatocellular carcinoma carcinogenesis by targeting CDK4. Int J Cancer. 2019;144:2489-2500. 30125344 10.1002/ijc.31816

[49] Siegel RL , Miller KD , Jemal A . Cancer statistics, 2019. CA Cancer J Clin. 2019;69(1):7‐34. 30620402 10.3322/caac.21551

[50] Wan J , Liu H , Feng Q , Liu J , Ming L . HOXB9 promotes endometrial cancer progression by targeting E2F3. Cell Death Dis. 2018;9:509. 29724991 10.1038/s41419-018-0556-3 PMC5938704

[51] Wan J , Liu H , Ming L . Lysine crotonylation is involved in hepatocellular carcinoma progression. Biomed Pharmacother. 2019;111:976‐982. 30841477 10.1016/j.biopha.2018.12.148

[52] Olsen CA . Expansion of the lysine acylation landscape. Angew Chem Int Ed Engl. 2012;51:3755‐3756. 22374739 10.1002/anie.201200316

[53] Yang XJ , Seto E . Lysine acetylation: codified crosstalk with other posttranslational modifications. Mol Cell. 2008;31:449‐461. 18722172 10.1016/j.molcel.2008.07.002 PMC2551738

[54] Millar AH , Heazlewood JL , Giglione C , Holdsworth MJ , Bachmair A , Schulze WX . The scope, functions, and dynamics of posttranslational protein modifications. Annu Rev Plant Biol. 2019;70(1):119‐151. 30786234 10.1146/annurev-arplant-050718-100211

